# Evaluation of a Home-Based Environmental and Educational Intervention to Improve Health in Vulnerable Households: Southeastern Pennsylvania Lead and Healthy Homes Program

**DOI:** 10.3390/ijerph13090900

**Published:** 2016-09-09

**Authors:** Deepa Mankikar, Carla Campbell, Rachael Greenberg

**Affiliations:** 1 Public Health Management Corporation, Centre Square East 1500 Market St., Philadelphia, PA 19102, USA; 2 Department of Public Health Sciences, University of Texas at El Paso, 500 W. University Ave., El Paso, TX 79968, USA; ccampbell3@utep.edu; 3 National Nurse-Led Care Consortium, Centre Square East 1500 Market St., Philadelphia, PA 19102, USA; rgreenberg@nncc.us

**Keywords:** environmental health, health disparities, housing, health education, childhood asthma, injury prevention, childhood lead poisoning, home health, home safety, healthy homes

## Abstract

This evaluation examined whether participation in a home-based environmental educational intervention would reduce exposure to health and safety hazards and asthma-related medical visits. The home intervention program focused on vulnerable, low-income households, where children had asthma, were at risk for lead poisoning, or faced multiple unsafe housing conditions. Home visitors conducted two home visits, two months apart, consisting of an environmental home assessment, Healthy Homes education, and distribution of Healthy Homes supplies. Measured outcomes included changes in participant knowledge and awareness of environmental home-based hazards, rate of children’s asthma-related medical use, and the presence of asthma triggers and safety hazards. Analysis of 2013–2014 baseline and post-intervention program data for a cohort of 150 families revealed a significantly lower three-month rate (*p* < 0.05) of children’s asthma-related doctor visits and hospital admissions at program completion. In addition, there were significantly reduced reports of the presence of home-based hazards, including basement or roof leaks (*p* = 0.011), plumbing leaks (*p* = 0.019), and use of an oven to heat the home (*p* < 0.001). Participants’ pre- and post- test scores showed significant improvement (*p* < 0.05) in knowledge and awareness of home hazards. Comprehensive home interventions may effectively reduce environmental home hazards and improve the health of asthmatic children in the short term.

## 1. Scope of Housing-Related Morbidities

Exposure to environmental home-based hazards, such as asthma triggers (e.g., mold, pests, secondhand smoke), lead, fire, and fall hazards, contributes to adverse health outcomes, including asthma, lead poisoning, and home injuries. Families of low socioeconomic status and racial/ethnic minorities are disproportionately exposed to environmental housing hazards and have an increased susceptibility to experiencing poor health outcomes upon exposure [1,2,3]. This is a significant environmental justice concern across communities.

Asthma affects one in every 15 individuals, and the prevalence has been increasing in recent decades [4]. Among children, asthma is the most common long-term disease, with urban environments posing an increased risk [4]. Trend analyses reveal prevailing disparities in asthma health outcomes; for instance, “low income populations, minorities, and children in inner cities experience more emergency department visits, hospitalizations, and deaths due to asthma than the general population” [5]. Secondhand smoke, an asthma trigger and significant health hazard, contributes to an estimated 202,000 childhood asthma exacerbations, 7330 lung cancer deaths, and 33,950 heart disease deaths annually [6]. In addition, children’s increased respiratory rate, smaller body size, and increased contact with indoor surfaces (e.g., carpets, furniture, floors, and smoke-infused clothing of caregivers) contribute to greater susceptibility for all forms of tobacco smoke exposure through inhalation and ingestion [7]. The 2006 U.S. Surgeon General’s report proclaimed that no level of tobacco smoke exposure is risk-free [8]. Since tobacco smoke can travel through home air systems and cracks in walls and floors, families of non-smokers are still exposed to smoke contamination from adjacent households with smokers [9]. This becomes a larger problem in multi-unit housing, which currently represents 26 percent (34.8 million) of all U.S. housing units [10].

In the U.S., the use of residential lead paint was banned in 1978; however, the presence of lead-based paint hazards is still troublesome. Based on sampling for the 2005–2006 American Healthy Homes Survey, an estimated one-third of all housing units contain lead-based paint (93 percent are pre-1978 built homes), and approximately 23.2 million homes have lead-based paint hazards, including deteriorating lead-based paint, lead dust, and lead in soil [11]. When children are exposed to lead through ingestion or inhalation of lead-contaminated house dust or ingestion of chipping and peeling lead paint, they are at risk for developing nervous system damage, including cognitive impairment, behavioral problems, and learning disabilities [12]. Disparities in lead exposure are also observed; lead-based paint hazards are more prevalent in low-income (compared to high-income) households and African American (compared to White) households [11]. A separate lead sampling data study, confirmed these findings, by concluding the year of home construction, income, and race as predictors of home lead exposure [13].

Each year, at least 30,000 fatal injuries and 12 million nonfatal injuries occur at home [14]. Falls, poisonings, fires, and burns are the leading causes of unintentional home injuries [15].

Falls among all ages often occur due to tripping down stairs or between floor levels and slipping in the bathtub. Among children, fall injuries may also result from tripping or climbing on furniture, falling from a window, and falling off a porch or deck [14,15]. Since only nonfatal fall injuries that require medical attention and fatal falls are documented, data on fall injuries are greatly underestimated. Some methods of prevention for home fall injuries include the use of gates at the top of stairs, window guards, grab bars on walls to prevent slipping in bathtubs, and handrails to prevent tripping down stairs [14,15].

While unintentional poisonings affect all age groups, the sources of poisonous substances differ between children and adults. Child poisonings commonly involve household products and adult poisonings commonly result from prescription drug overdose [14]. Some household products subject to unintentional poisonings include “personal care products, household cleaners, pesticides, and medications” [14]. Bleach is the most recognized household cleaner capable of producing toxic effects and should be used cautiously [14]. As a respiratory irritant, bleach is also a concern for asthmatics; previous occupational health studies reveal an association between bleach exposure and asthma symptoms [16,17]. To prevent unintentional poisonings that occur from ingesting household products or medicines, one should place all potentially dangerous household products and medicines in a cabinet or drawer that is high up, locked, and out of a child’s reach.

Carbon monoxide (CO) poisoning occurs through inhalation of CO gas, which is “produced from incomplete combustion of carbon-containing substances” [14]. Sources that produce CO gas include fires, appliances that run on gas (e.g., space heaters, stoves, furnaces, generators, and fireplaces), and vehicle exhaust [14]. Since one cannot see nor smell CO gas, the effects can be life-threatening with a long duration of exposure. Non-fire related unintentional CO poisonings account for 400 deaths, 20,000 emergency department visits, and 4000 hospitalizations every year [18]. Carbon monoxide poisoning can be prevented by ensuring proper ventilation when using gas appliances and by using a CO detector [14].

Every year, house fires result in approximately 2560 deaths and 13,000 injuries [15]. While cooking is the most common source of house fires, smoking-related fires cause the most deaths [14]. Burn injuries are caused by fires, electric shocks, scalds from hot liquids, and contact with hot surfaces [14]. Negative health impacts of fire and burn injuries are characterized by adverse respiratory effects or tissue damage [14]. Fire- and burn-related injuries and deaths can be prevented by installing working smoke detectors and minimizing contact with sources of electricity, heat, and open flames.

### 1.1. Pennsylvania Health Disparities

Southeastern Pennsylvania is a focus of public health prevention work due to the population’s socioeconomic and housing disparities, which increase susceptibility to poor health outcomes. Over half of all Philadelphia rental homes contain at least one housing hazard, such as “open cracks or holes in walls and floors, mice, and water leaks from inside” [19]. Among all Pennsylvanian counties, Philadelphia County has the largest percent of families with children living below the poverty level (26%), which is double the statewide estimate (13%) [20]. Additionally, in the past decade, every minority population group in Pennsylvania increased in size [20]. According to the Pennsylvania Department of Health, asthma prevalence was greater among non-Hispanic African American adults than non-Hispanic white adults (13% to 9%, respectively) in 2012 [21]. Similar trends for asthma prevalence were noted among non-Hispanic African American children compared to non-Hispanic white children (13% to 8%, respectively) [21].

### 1.2. Evidence Supporting Comprehensive Home Interventions

In-home prevention education is one way to inform families about sources of environmental hazards and methods to reduce exposure. Previous home-based environmental education programs improved client knowledge of environmental exposures and reduced the presence of home hazards by using community health workers (CHWs) to deliver health education and other services to low-income, vulnerable populations [22,23,24]. The American Public Health Association defines a CHW as a “frontline public health worker who is a trusted member of and/or has an unusually close understanding of the community served” [25]. Through this relationship, the community health worker improves an individual’s access to health education, social support, and healthcare services, while ensuring the delivery of culturally-competent care [25]. Viswanathan and colleagues conducted a review of CHW interventions for vulnerable populations that revealed outcomes of improved knowledge, as well as behavior changes for better asthma management [26].

In the Seattle Healthy Homes Project, 274 households from King County, Washington were randomly assigned to a “high-intensity” or “low-intensity” asthma intervention [23]. The participants were low-income families with children who have asthma. Those in the high-intensity intervention received an environmental home assessment, education by CHWs, help to create an asthma action plan, and resources (e.g., pillow and mattress covers, cleaning supplies, and referrals to smoking cessation programs) over multiple home visits throughout one year. Those in the low-intensity intervention received one home visit by a CHW, during which limited education and supplies were given and an action plan was discussed, followed by additional education and resources a year after program completion. Krieger and colleagues concluded that the high-intensity intervention, which consisted of a multi-faceted approach, was more successful than the low-intensity intervention at reducing the use of urgent care services, reducing asthma symptom days, and increasing caregiver quality of life scores [23]. Furthermore, in comparison to a low-intensity group participant, a high-intensity group participant would save an estimated $189–$721 more in four years from reductions in urgent care use, including “hospital admissions, emergency department visits, and unscheduled clinic visits” [23].

In the Philadelphia Lead Safe Homes Study, primary prevention methods addressed lead exposure in the homes of families with newborns. Through the use of education, home assessment, and lead remediation, families significantly improved their cleaning practices, as well as their knowledge on lead exposure [22]. Over the twelve month study period, a reduction in lead dust exposure was observed through a significant decrease in positive dust wipe samples from windowsills [22]. While it may be difficult to assess the cost savings provided by education on home lead hazards, if parental education leads to better understanding and use of effective cleaning practices, one can infer that improved housekeeping will help reduce lead dust exposure and the risk of developing health effects from elevated blood lead levels.

### 1.3. Healthy Homes

In an effort to meet the Healthy People 2020 goal of improved home environmental conditions, the Healthy Homes model is promoted through various U.S. government agencies, such as the Centers for Disease Control and Prevention, the Environmental Protection Agency, and the Department of Housing and Urban Development. These agencies provide materials and support trainings to help states and non-governmental organizations develop their own Healthy Homes programs [27]. An example of one of these programs is the Southeastern Pennsylvania Lead and Healthy Homes Program (SPLHHP). By offering free in-home assessments, education on home-based environmental hazards, and supplies to maintain a healthy home environment, the SPLHHP is designed to help vulnerable populations access resources and guidance to achieve healthier and safer homes. Although the SPLHHP was not designed as a formal study, the program implements home-intervention components used in the above-mentioned studies. Thus, the potential effectiveness of the SPLHHP in addressing home hazards is worth exploring.

Through the following program evaluation, the authors aim to:

- Analyze whether there is an increase in knowledge and awareness regarding home-based environmental hazards, as determined by comparison of data from Part I of the pre-EHA questionnaire and post-EHA questionnaire;
- Analyze whether there is a decrease in client-reported medical visits for children with asthma, as determined by comparison of data from Part II of the pre-EHA questionnaire and post-EHA questionnaire; and
- Assess whether there is a decrease in client-reported presence of health and safety hazards, as determined by comparison of data from Part III of the pre-EHA questionnaire and post-EHA questionnaire.

Program evaluation was approved by the Drexel University Institutional Review Board (project ID 1409003123). The results of this program evaluation can inform public health professionals on the possible effectiveness of comprehensive home intervention methods (e.g., environmental assessment, in-home education, Healthy Homes supplies, and referrals) in reducing environmental hazard exposure, improving the health of vulnerable populations, and increasing environmental justice.

## 2. Program Description

The SPLHHP is a primary prevention home-intervention program, designed with the aim of reducing adverse health effects associated with environmental home hazards, especially those experienced by children. The program is funded by the Pennsylvania Department of Health (through a federal Health Resources and Services Administration Maternal and Child Health Services Block Grant), and run by the National Nurse-Led Care Consortium (NNCC), a non-profit organization serving communities through nurse-led healthcare and community health programs. The program consists of two home visits that take place two months apart. The initial home visit is scheduled to last approximately two hours. The final home visit is shorter, lasting approximately one hour. Home intervention services are delivered by a home visitor, who is either a CHW, an environmental health professional, or a nurse. All home visitors are trained in Healthy Homes principles prior to starting the program. Program services include home assessment, environmental education, and distribution of supplies and referrals, as needed. While the program provides education on lead poisoning prevention, asthma prevention and management, and injury prevention, the intervention is largely focused on the reduction of asthma triggers and safety hazards. Over the program’s thee-year period, program providers strive to reach 1200 households across eight Southeastern Pennsylvania counties: Berks, Bucks, Chester, Delaware, Lancaster, Montgomery, Philadelphia, and Schuylkill [28]. First-year program data were collected from July 2013 to June 2014. Program evaluation took place between October 2014 and May 2015.

### 2.1. Program Components

#### 2.1.1. Healthy Homes Assessment

The Pennsylvania Department of Health created an Environmental Home Assessment (EHA) form with accompanying pre-/post- questionnaires for program use. At the initial home visit, the home visitor administers the pre-EHA questionnaire, which is used to collect information on demographics, asthma-related medical visits, knowledge of home-based hazards, and the presence of environmental hazards in the home. To accompany the pre-EHA questionnaire, the home visitor completes the EHA form with detailed information of the household conditions (e.g., home ventilation, location of indoor pollutants, housekeeping practices, condition of exterior/interior steps and railings, and storage of hazardous household supplies and medicines). When appropriate, the home visitor may refer participants to primary care providers for a blood lead test or further evaluation of asthma symptoms. Families may also be referred to appropriate social service programs, including: food pantries, early intervention programs, maternal and child health programs, benefit programs, code enforcement, and weatherization programs.

At the two month follow-up visit, the home visitor administers the post-EHA questionnaire, including the same questions from the pre-EHA on asthma-related medical visits, participant knowledge and awareness of home hazards, and some additional questions that describe the presence of exhaust fans, moisture, and carpeting.

In addition to the Healthy Homes assessment, an Environmental Inspection is offered to households with a child known to have elevated blood lead levels. This service is performed by a certified individual, and includes testing surfaces in a home to determine the presence of lead paint, visual identification of chipping or peeling paint, and collection and analysis of lead dust wipes or soil samples. The family receives an environmental inspection report, which includes a summary of the findings and recommendations to remediate lead hazards.

#### 2.1.2. In-Home Education

The home visitor provides education on ways to maintain a healthy home, through use of the “Help Yourself to a Healthy Home” educational booklet (created by the U.S. Department of Housing and Urban Development and U.S. Department of Agriculture). This booklet presents an overview of home hazards, including poor indoor air quality, mold, moisture, carbon monoxide, lead, household cleaning products, pesticides, and injury prevention concerns (i.e., slips, trips, falls, drownings, unintentional poisonings, fires, burns, and choking and suffocation hazards). Each section describes the hazard, the associated negative health outcomes, a set of action steps, and resources (online websites or organizational phone numbers) to obtain additional information. In addition, families receive educational materials that are specific to the hazards present in their households. For example, a family with household mold may receive “A Brief Guide to Mold, Moisture and Your Home”, developed by the U.S. Environmental Protection Agency [29]. The educational booklet is offered in English and Spanish, and all materials are designed to be sensitive to families with low-literacy skills. When providing educational tools to the family, the home visitor explains the hazards present in the family’s home and reviews strategies on how to resolve the problems.

#### 2.1.3. Healthy Homes Supplies

At the initial and follow-up visits, participants receive free supplies to aid in household cleaning (e.g., all-purpose cleaner, trash bags, and microfiber flip mop), pest management (e.g., roach baits, mouse glue traps, and mattress and pillow covers), and safety (e.g., smoke detector and carbon monoxide detector) (Table 1). Using these supplies, the home visitor teaches the family how to maintain a clean home environment (e.g., wet dusting and wet mopping vs. dry sweeping), manage pests (e.g., the home visitor explains best practices on how to set up roach bait stations and use mouse glue traps), and manage asthma (e.g., proper use of mattress and pillow covers).

### 2.2. Participant Criteria and Recruitment

To be eligible for the SPLHHP, families had to reside in Southeastern Pennsylvania and had an annual income that was below 250% of the 2013 Federal Poverty Level (The Federal Poverty Level [FPL] is a measure used to determine a familiy’s eligibility for government assistance programs, as well as other community health programs. The FPL is updated annually, and the calculation is based on household income and family size). Eligible families also had either an expecting mother or a child under 18 who had asthma, was at risk for lead poisoning (i.e., lived in a household built before 1978 with chipping or peeling paint), or self-reported another home health hazard (e.g., pests, mold, or injury prevention concerns). Environmental Inspection services were available for any child under seven years of age with an elevated blood lead level (defined as one confirmed blood lead level ≥20 μg/dL or two blood lead levels ≥15 μg/dL taken three months apart; in Philadelphia, an elevated blood lead level is defined as two blood lead levels ≥10 μg/dL taken three months apart). These inclusion criteria were determined as a means of increasing services to low-income families, while also targeting individuals who are the most at risk for adverse housing-related health outcomes.

Participants were recruited through self-referral and provider referral (e.g., physicians and community-based organizations). For provider referrals, the client signed a consent form at the provider’s office. By signing this form, the client agreed with the referral and waited to hear from SPLHHP staff for further instructions on enrollment in the program. Self-referred participants followed a similar enrollment process over the phone with SPLHHP staff. Once accepted into the program, all clients completed a detailed consent form at the initial home visit.

### 2.3. Sample Size

A sample size of 128 was deemed necessary based on the assumption that a 0.5 mean difference (SD = 2) in client-reported asthma medical visits at initial and follow-up visits would be observed. This sample size would allow for 80% power, given α = 0.05 (two-sided). These parameters were determined through comparison of similar home intervention studies, which looked at changes in “urgent health care events” and “self-reported emergency department visits and hospitalizations” [23,30]. Additionally, it was determined that a sample size of 128 will allow one to observe a mean difference of 1 (SD = 4) in participant scores for the knowledge and awareness questionnaire, with 80% power.

### 2.4. Data and Analysis

The NNCC compiled de-identified participant data from all program partners who conducted home visits (Program partners that contributed data include Chester County Health Department, Family Practice and Counseling Network, Montgomery County Health Department, National Nurse-Led Care Consortium, Pinnacle Health, and Temple Health Connection). Some regions did not adequately provide NNCC with all home assessment data; therefore, analysis was focused on data from the pre- and post-EHA questionnaires.

Halfway through the first program year, the pre- and post-EHA questionnaires were updated to improve question clarity and interpretation. The updated questionnaires were gradually implemented by all program partners. Parts of the updated questionnaires were not comparable to the original versions. Therefore, the following analysis is limited to households who completed the original pre- and post- questionnaires. The data can be made available upon request.

For the purpose of this evaluation, data from the first three sections of the pre- and post-EHA questionnaires were analyzed:

- Respondent’s Background Data
  The first section included descriptive information such as demographics, type of home, number of asthmatic children, and client reports of their children’s medical visits due to asthma. Clients were asked to recall the frequency of their children’s asthma-related doctor visits, emergency room visits, and hospital admissions within the past twelve months.
- Current Awareness/Knowledge
  The second section evaluated the participant’s knowledge and awareness of home-based environmental hazards through nine multiple-choice questions covering the topics of asthma triggers and home injury prevention. Participants were scored on the number of questions correctly answered. The following are some examples of these questions:
  *Which of the following are sources of CO in the home?* *(there can be more than one answer)*
  Answer choices: space heater; stove; cigarette smoke; steam from the shower; does not know
  How often should you test your smoke alarm battery?
  Answer choices: one time per year; one time per month; once every two years; does not know
  Which of the following can trigger asthma flare-ups? (there can be more than one answer)
  Answer choices: Steam-cleaning the carpet; drinking milk; pet dander; pests; does not know
- Observation and Questionnaire
  The third section included observations on home conditions, ranging from presence of asthma triggers (e.g., pets, pests, and mold) to safety hazards (e.g., presence of a CO detector and use of an oven to heat the home).

The evaluation includes a descriptive analysis of all valid first-year program data (2013–2014) and comparative analyses for measures assessed at both initial and final home visits. These measures include the mean difference in knowledge scores from pre- and post-EHA questionnaires, client-reported changes in the presence of home-based hazards, as well as client-reported changes in the rate of medical visits for their children with asthma. Program data were managed in Microsoft Excel 2010 (Microsoft Corporation, Redmond, WA, USA). All analyses were conducted in SPSS Version 20.0 (IBM Corporation, Armonk, NY, USA).

## 3. Results

### 3.1. Participant Demographics and Characteristics

In the first program year, 283 families completed an initial home visit. Of this group, 150 families completed a two-month follow-up visit (rate of follow-up = 53%). Individuals who did not have data from the final visit were either lost to follow-up or received the final visit in the following program year. Factors that contributed to loss to follow-up included non-response to contact attempts (by phone and in person) made by the home visitor, scheduling conflicts, and changes in client’s phone-number or home address.

The majority of program participants identified themselves as African-American (70.6%), Hispanic (56.5%), and/or Caucasian (18.1%), with few identifying as other racial and ethnic groups (this included allowances for identifying with multiple categories). Only small differences exist in the 150 case sample that completed both home visits within the program year (Table 2).

In total, there were 651 children. The number of children in a household ranged from 1–9 (Mean = 2.4, SD = 1.44). The prevalence of client-reported asthma among children was 28 percent. Among the 183 children with asthma, 60 percent were between ages 1–5, 35 percent were between ages 6–17, and five percent were less than one year of age (Table 2). 

More than half of all program participants lived in row/duplex housing (59.3%), followed by apartments (22.8%) and single detached homes (16.3%) (Table 2). Philadelphia County comprised the greatest proportion of all participating households (61.1% overall, and 64.0% of the follow-up sample).

### 3.2. Asthma-Related Medical Use

Childrens’ asthma-related medical use data, reported by participants at initial and final visits, were analyzed as three-month rates using the Wilcoxon Signed Rank test. Findings revealed a significantly lower rate of hospital admissions (Z = −4.639, *p* < 0.001) and doctor visits (Z = −2.579, *p* = 0.010) reported at the final visit compared to the initial visit. There was also a lower, but nonsignificant, rate of emergency department visits (Z = −1.777, *p* = 0.076) reported at the final visit.

### 3.3. Home-Based Health and Safety Hazards

#### 3.3.1. Presence of Hazards

**Figure 1:**
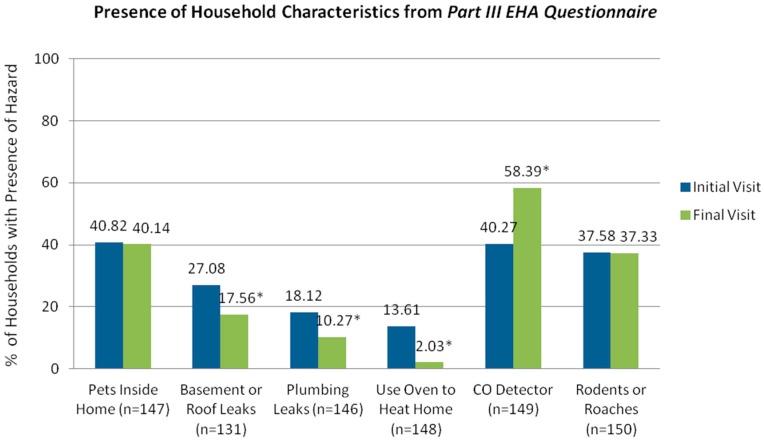
Presence of household hazards at initial and final visits.* *p <* 0.05.

Compared to client reports at the initial visit, follow-up reports revealed reductions in the presence of indoor pets, pests, and basement or roof leaks, as well as self-reported use of an oven to heat the home. McNemar tests indicated significant changes in the proportion of clients reporting the presence of certain home-based hazards (Figure 1), including basement or roof leaks (*p* = 0.011), plumbing leaks (*p* = 0.019), use of an oven to heat the home (*p* < 0.001), and a working carbon monoxide detector (*p* < 0.001). Additional home surveyor observations, recorded only during the final visit, revealed health hazards that contribute to moisture accumulation and mold growth; among all surveyed homes, 53 percent had kitchen exhaust fan ventilation, while fewer households had bathroom exhaust ventilation (46%), carpeting in every room (17%), and a musty/moldy smell (9%).

#### 3.3.2. Knowledge and Awareness of Hazards

**Figure 2:**
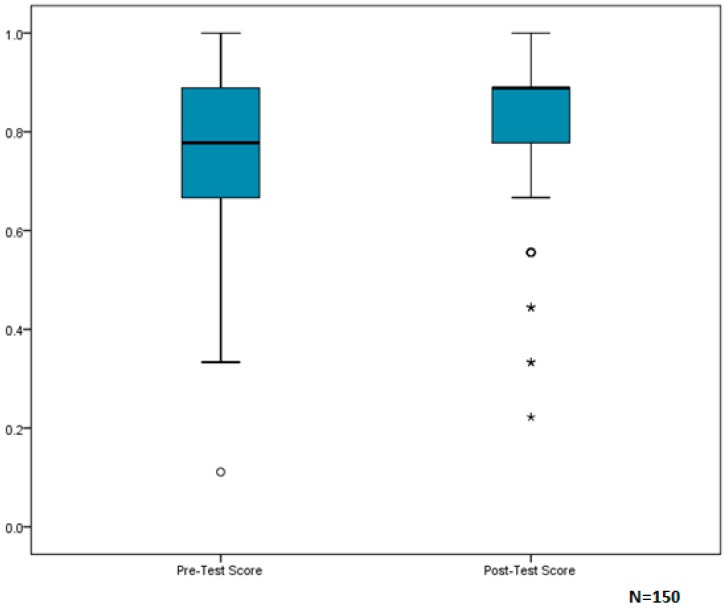
Box-and-whisker plots of participant median scores on Part II of the EHA Questionnaire: Awareness/Knowledge.

For assessment of current knowledge and awareness of home-based hazards, paired *t*-test analysis indicated that participants had a mean score of 78/100 on the pre-test, and showed significant improvement (*p* = 0.008, 95% CI (0.01, 0.07)) on the post-test with a mean score of 82/100. Due to the slightly greater negative skew in post-test scores, results were also checked using a nonparametric test. A Wilcoxon Signed Rank Test confirmed significant improvement between pre- and post-test scores (*p* = 0.007) (Figure 2).

**Figure 3:**
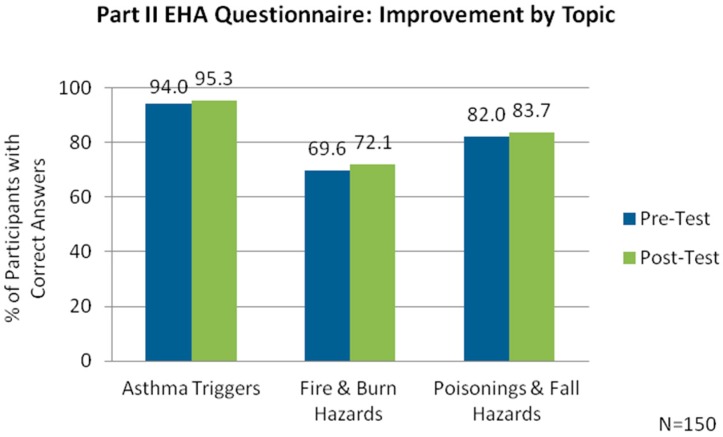
Assessment of participants’ awareness and knowledge of home hazards by topic.

Participant scores were also assessed with the questions grouped by topic. The scores indicated improved knowledge across all topics, with the greatest improvement for questions on fire and burn hazards (Figure 3).

## 4. Discussion

### 4.1. Discussion and Contribution to Literature

These findings support the usefulness of home-based interventions in improving the health and home conditions of a vulnerable population. The Healthy Homes education, delivered by the home visitor, was effective in increasing clients’ knowledge and awareness of these hazards, as seen by the significant improvement in scores between pre- and post- EHA questionnaires (Figure 2). Additionally, educating clients on methods to reduce home-based hazards and providing the necessary supplies to engage in home safety and housekeeping practices may have resulted in improved home conditions. One may speculate that improvements in the home environment contributed to the reduced rate of asthma-related doctor visits and hospital admissions.

Previous studies of educational asthma home interventions have shown similar outcomes. In a West Philadelphia study of asthmatic children, families who received in-home education by CHWs on asthma triggers, asthma self-management techniques, and methods to reduce asthma triggers (e.g., supplies and/or home remediation), experienced fewer asthma-related inpatient hospitalizations, emergency department visits, and primary care visits compared to a control group that received no in-home intervention [31]. A larger inner-city asthma study, which also implemented education and an environmental home assessment, found an association between reduced allergens and decreased asthma morbidity (e.g., hospitalizations and unscheduled medical visits due to asthma) in the intervention group [32]. Findings from the SPLHHP analysis also confirmed the difficulty of reducing certain asthma triggers (e.g., pets and pests inside the home) among vulnerable populations, as observed in prior studies [33,34].

### 4.2. Limitations

Client-reported information on asthma-related medical visits was subject to differential recall bias, with a stronger effect on pre-EHA responses, as the pre-EHA questionnaire asked the participant to recall events within the past year, in contrast with the post-EHA questionnaire which had varying reporting intervals (e.g., emergency room visits and hospital admissions within the past three months; doctor visits within the past year). Although analyses were adjusted by converting all data to reflect a rate over a three-month period, the comparison of these medical events was somewhat hindered by these inconsistencies.

Time constraints and limited staffing for data entry and analysis made it difficult to fully utilize the EHA form data. This form contains detailed housing information on a room-by-room basis, and the analysis of such information may have provided better insight into home hazards that were unique to each child’s environment (e.g., asthma triggers in a child’s sleeping environment).

A commonly cited possible phenomenon with home interventions is the Hawthorne Effect. This theory says improvement in an individual’s behavior or actions is influenced by the knowledge that one is being observed [35]. Therefore, it is possible that participants maintained housekeeping practices between the initial and final visit due to knowledge that they would be observed and evaluated for changes in the presence of home hazards.

The SPLHHP experienced difficulties with follow-up, similarly described in other home intervention programs serving vulnerable populations [22,36]. Despite the relatively short time between intervention and reassessment, loss to follow-up often occurred due to difficulties with phone contact. Zook et al. describe common challenges with establishing phone contact, characteristic of low-income populations, which include disconnected phone service, frequent changes in cell phone carrier, and limited minutes with cell phone plans [37]. The follow-up rate of 53 percent may have led to a biased sample, as those who completed both home visits may have been more likely to comply with Healthy Homes recommendations and score higher on the knowledge assessment.

### 4.3. Future Research and Recommendations

The SPLHHP was successful in its ability to address environmental home hazards in a vulnerable population. By using educational materials that were prepared by public health government entities, the information presented on home-based hazards is standardized to other programs that address creating and maintaining healthy homes. Additionally, the educational materials and teaching methods are sensitive to low literacy levels and cultural differences, making them generalizable for use in low-income neighborhoods that reflect the demographics of Southeastern Pennsylvania. More specifically, this evaluation will benefit other environmental home intervention programs, as it identifies areas to improve in-home education (e.g., more clarification for carbon monoxide sources in the home and greater inclusion of lead topics).

The program’s unique funding source, a Maternal and Child Health Services Block Grant, may provide insight to other community-based organizations on available resources to consider when planning a Healthy Homes program.

For future implementation of Healthy Homes programs, the following improvements in tools and program components may enable a more robust evaluation. The pre- and post-EHA questionnaires should have greater question clarity (e.g., accuracy of answer choices) and consistency (e.g., recall of past medical events), which will help to more reliably characterize and analyze client knowledge and awareness of home hazards, as well as client-reported asthma medical visits. Additionally, the use of a medical event diary, completed during the program, may provide better accuracy of client-reported data. Asthma diaries, a self-reporting tool used in previous asthma management home intervention programs, helped to assess changes in symptoms [33,38] and medication use [33]. While the home visitors noted supplies to bring to the follow-up visit, a personalized asthma action plan may provide families with home-specific recommendations to reduce asthma triggers and manage their child’s asthma. To make this plan accessible for families, program providers should form a partnership with clinicians who already serve children in the communities targeted by the program. A longer program period between intervention and reassessment should also be considered. This would allow one to better assess the retention of acquired healthy homes knowledge and provide more time to observe reductions in the presence of certain asthma triggers, such as pests. Additionally, hospital admissions and medical visits are relatively infrequent events; therefore, a longer reporting period might lead to better knowledge regarding subsequent asthma-related medical visits.

Breysse and colleagues conducted a study comparing group homes that received in-home asthma education by CHWs and structural weatherization-plus-health interventions to a historical sample that only received in-home asthma education [39]. The homes that received the combined education and weatherization services showed significant improvement in children’s asthma control, along with improved caregiver quality of life measures [39]. Although the SPLHHP refers families to weatherization programs, it may be beneficial for future Healthy Homes programs and weatherization programs to combine services to more effectively serve families with asthmatic children.

The success of the SPLHHP was greatly due to the range of services provided by the home visitors, who delivered health education, as well as assessments for housing and health. While the home visitors represented different professions, all were trained in Healthy Homes principles, and were therefore effective in providing program services. Others looking to implement a Healthy Homes program should consider employing health professionals and CHWs to deliver the interventions. When serving disadvantaged populations, public health interventions should incorporate a diverse range of services and areas of expertise to meet family-specific service needs.

To more vigorously assess program effectiveness, future research on Healthy Homes programs should involve either a case-control study design or cohort study design that includes all the program components and recommendations described above. A case-control study can help determine whether improved outcomes are attributable to the intervention services. With sufficient time and funding, a cohort design can provide valuable insight into the long-term outcomes of home intervention services.

### 4.4. Implications for Environmental Justice

The negative health impact, resulting from socioeconomic and health disparities, is pronounced in low-income families of racial/ethnic minority groups. Individuals from these vulnerable groups are greatly affected by asthma triggers, lead exposure, and safety hazards present in their homes. Implementing a program within these communities to address the home environment—a critical and often overlooked component of their health—can have significant implications for improving environmental justice and alleviating a portion of these poor health outcomes.

The SPLHHP empowers families to create healthier and safer housing conditions by providing health education and resources to reduce exposure to home health hazards, thereby reducing their susceptibility to housing-related morbidities. The program also demonstrates the feasibility of promoting household-level changes to modify indoor environmental factors that contribute to health disparities in vulnerable communities. However, to truly reduce environmental health disparities, home interventions must be accompanied by community-level action that targets external environmental factors related to neighborhood infrastructure. More specifically, public health officials, land use planners, local government officials, and other community stakeholders should work together to improve the social and physical neighborhood conditions in disadvantaged communities. This may involve plans to identify and minimize neighborhood stressors, enforce neighborhood safety measures, reduce outdoor sources of air pollution, improve access to public transportation and community facilities, and provide resources for housing stability.

## 5. Conclusions

Adverse health effects from poor housing conditions continue to burden vulnerable populations. As previous studies demonstrate, primary prevention home intervention programs are successful in equipping families with the knowledge, supplies, and resources to reduce environmental home-based hazards and improve the health of asthmatic children. Although there are areas for program improvement, first-year analysis of the SPLHHP revealed promising short-term findings, including significant improvement in awareness and knowledge of home hazards, significant reduction in rates of asthma-related hospital admissions and doctor visits, as well as significant reductions in the reported presence of numerous home-based hazards. Through consideration and implementation of the above-mentioned recommendations in future Healthy Homes programs, public health organizations can reduce the negative health effects of environmental housing disparities and provide all families the opportunity to create and maintain healthy homes.

## Figures and Tables

**Table 1:** Supplies available for distribution to SPLHHP households.

**Cleaning**	Dual Action microfiber flip mop, microfiber cloth, all-purpose cleaner, gloves, hand soap, trash bags, trash can, and storage bin.
**Safety**	Smoke detector, carbon monoxide detector, electrical outlet covers, and night light.
**Pest & Asthma Management**	Roach baits, mouse glue traps, caulk, caulk gun, pillow covers, and mattress covers.

**Table 2:** Participant demographics and household characteristics.

Characteristic	Total Sample N (%)	Follow-Up Sample N (%)
Total Cases	283	150
*County Representation*		
Philadelphia	173 (61.1)	96 (64.0)
Chester	27 (9.5)	22 (14.7)
Montgomery	46 (16.3)	10 (6.7)
Berks, Bucks, Schuylkill	37 (13.1)	22 (14.7)
*Type of Home*		
Apartment	60 (22.8)	34 (24.1)
Manufactured	4 (1.5)	3 (2.1)
Row/Duplex	156 (59.3)	75 (53.2)
Single Detached	43 (16.3)	29 (20.6)
*Race/Ethnicity* ^1^		
African-American	168 (70.6)	88 (67.7)
Asian	2 (0.8)	1 (0.8)
Caucasian	43 (18.1)	27 (20.8)
Hispanic ^2^	74 (56.5)	39 (58.2)
Native American	1 (0.4)	1 (0.8)
Pacific Islander	3 (1.3)	2 (1.5)
More than one race	7 (2.9)	2 (1.5)
Other	14 (5.9)	9 (6.9)
Total Children	651	359
*Children with Asthma*	183 (28.0)	118 (32.8)
Less than 1 years old	10 (5.5)	7 (5.9)
Between ages 1–5	109 (59.6)	68 (57.6)
Between ages 6–17	64 (34.9)	43 (36.4)

^1^ Race/ethnicity classification includes multiple responses; totals exceed 100%; ^2^ Hispanic classification is inclusive of Cuban, Latino, Mexican, and Puerto Rican ethnic groups.

## Abbreviations

The following abbreviations are used in this manuscript:

SPLHHP	Southeastern Pennsylvania Lead and Healthy Homes Program
DOAJ	Directory of open access journals
MDPI	Multidisciplinary Digital Publishing Institute
NNCC	National Nurse-Led Care Consortium
CHW	Community Health Worker
EHA	Environmental Home Assessment
